# Effects of mechanical and electrical stimulation on accelerating greenstick fracture Healing: Insights from finite element analysis and experimental validation

**DOI:** 10.1016/j.jor.2025.07.011

**Published:** 2025-07-15

**Authors:** Mohamed Hassan, Enas Fawzi Youssef, Ahmed Rizk Mohamed, A.R. El-Dhaba, Mohamed I. Zineldin, Dina S. Abd Allah

**Affiliations:** a Department of Engineering, School of Computing and Engineering, University of Huddersfield, United Kingdom; b Department of Physical Therapy for Musculoskeletal Disorders and its Surgery, Faculty of Physical Therapy, Cairo University, Egypt; c Faculty of Medicine, Cairo University, Egypt; d Department of Mathematics and Statistics, College of Science, King Faisal University, P.O. Box 400, Al-Ahsa, 31982, Saudi Arabia; e Department of Mathematics, Faculty of Science, Damanhour University, P.O. Box 22511, Damanhour, El behira, Egypt; f Faculty of Engineering, Menoufia University, Egypt

**Keywords:** Greenstick fracture, Finite element analysis (FEA), Brachioradialis muscle, Flexoelectric effect

## Abstract

**Background:**

Greenstick fractures are common forearm injuries in children, with 75–84 % occurring in the distal third of the radius. Conservative treatments such as detachable braces or plaster backslabs permit early physiotherapy intervention, including muscle activation exercises and electrotherapy. This study investigated the biomechanical and electrophysiological effects on fracture healing to improve bone strength and reduce refracture risk, using finite element analysis (FEA) and experimental validation.

**Objective:**

To evaluate the effect of controlled isometric muscle contractions and localized electrical stimulation on fracture healing in pediatric distal radius greenstick fractures, integrating computational modeling with experimental case validation.

**Participants and setting:**

The experimental component involved two pediatric patients (aged 8–10 years) diagnosed with distal third greenstick fractures of the radius. Clinical management and data collection were conducted in a physiotherapy outpatient department at a tertiary care hospital.

**Methods:**

A 3D model of a radius bone with a distal third greenstick fracture was developed. FEA using ANSYS analyzed strain distribution under isometric contraction of the brachioradialis muscle, with repetitions ranging from 5 to 30. COMSOL Multiphysics also simulated electrical stimulation by applying a 6.25 V potential across the fracture site, assessing displacement and strain alterations. Experimental validation included two pediatric cases: Child A received standard conservative treatment with immobilization, while Child B received local electrical stimulation. Healing progression was quantified by measuring fracture gap reduction by MATLAB-based image analysis.

**Results:**

ANSYS simulations indicated that low-repetition (3–5) isometric muscle contractions may enhance callus formation. COMSOL simulations demonstrated a low strain gradient with electrical stimulation, but experimental validation showed a significant 58.8 % reduction in fracture gap area using electric stimulation, compared to 31.8 % with conservative treatment.

**Conclusions:**

Applying controlled isometric mechanical loading and electrical stimulation may reduce the fracture gap in greenstick distal radius fractures, accelerate healing and recovery. Future studies should explore direct current electrical stimulation (DCES) for potentially more robust effects on bone remodeling.

## 1 Introduction

Fractures of the distal end of the radius are among the most common injuries in children, accounting for a significant proportion of all pediatric fractures.^1^ These injuries typically result from falling on an outstretched hand, occurring during everyday activities or sports. Due to the skeletal immaturity of children, these fractures are often present as greenstick fractures, which are partial fractures.^2^

A greenstick fracture is characterized by interruption and deformation of the cortex and periosteum on one side of the bone, while the other side remains intact. This unique presentation is due to the higher proportion of collagen and greater flexibility in immature bones, which predisposes them to bending rather than complete fracture.^3^ Despite the partial nature of these fractures, improper management could lead to complications such as recurrent forearm fractures, highlighting the need for a well-planned therapeutic approach.^4^ The management of greenstick fractures is predominantly conservative, with the choice of immobilization methods being guided by the degree of angulation.^5^ For children younger than 10 years, minimal angulation (≤15°) can be effectively managed with a short-arm splint and six weeks of immobilization. More pronounced angulation (>15°) often requires the use of a soft cast to maintain alignment and promote healing.^6^ However, despite appropriate management, 6.7 % of patients experienced a refracture within a median of 49 days after plaster removal, highlighting the need for careful follow-up to prevent complications.^7^

Physical therapy plays a pivotal role in the recovery phase, focusing on restoring range of motion, improving muscle strength, and alleviating pain.^8^, ^9^, ^10^ Despite this, limited attention has been given to applying physical therapy modalities during the bone healing phase, particularly in identifying the specific mechanical parameters that can trigger, enhance, and accelerate the bone repair process.^11^

Mechanical loading plays a crucial role in stimulating cellular communication and triggering tissue proliferation for effective healing. According to strain theory, mechanical strains within the range of 2 %–10 % are optimal for bone formation. Strains outside this range either too low or too high can impair the healing process or delay recovery.^12^

Muscle-generated forces, especially from the brachioradialis muscle is uniquely positioned to contribute to fracture stability due to its horizontal force component, which aids in maintaining alignment and counteracting lateral deviations.^13^ Moreover, it may provide controlled mechanical loading along the bone's axis, promoting optimal conditions for bone healing and remodeling, accelerate healing by promoting optimal inter-fragmentary motion and reducing the risk of delayed union or malunion.^14^

In addition to mechanical forces, studies have shown that electrical stimulation in pediatric populations has been extensively investigated across various clinical conditions, indicating a strong safety profile. Specifically, modalities such as Neuromuscular Electrical Stimulation (NMES) and Functional Electrical Stimulation (FES) were shown to be safe and well-tolerated in children, with no significant reports of harmful effects.^15^ It has been shown that electrical stimulation enhances cell migration, increase mineralization, and activate osteogenic genes, all of which are critical for bone repair.^16^, ^17^ The piezoelectric and flexoelectric properties of bone materials, such as hydroxyapatite, play a pivotal role in this process. Piezoelectricity refers to electrical charge generation in response to uniform mechanical deformation, while flexoelectricity involves electric polarization induced by a strain gradient (non-uniform deformation), such as at fracture edges or crack tips. Both effects stimulate osteocyte activity and promoting bone healing.^18^, ^19^ Additionally, converse flexoelectricity, defined as the mechanical stress induced by an electric polarization gradient, is anticipated to enhance healing through electrical stimulation. By generating mechanical strains, this phenomenon activates osteocytes, further emphasizing its therapeutic potential in facilitating bone repair.^20^ The hydroxyapatite in the cortex layer of bone makes this mechanism especially relevant for greenstick fractures, where partial cortical disruption aligns well with the flexoelectricity theory.^19^

Despite the theoretical benefits of these mechanisms, their clinical application has been inconsistent due to variability in treatment parameters and the lack of evidence supporting their efficacy.^21^ To address these challenges, computational modeling techniques, such as finite element analysis (FEA), provide a powerful platform to simulate the biomechanical environment and predict fracture-healing behavior under different conditions.^22^ FEA has been successfully applied in various contexts, including estimating interfragmentary movements,^23^ evaluating the effects of loading conditions,^24^ and recommending patient-specific therapeutic interventions.^25^ So, this study aimed to evaluate the biomechanical and electrophysiological effects of applied controlled brachioradialis muscle force and electrical stimulation on greenstick distal third of the radius fracture healing using finite element analysis and experimental validation.

This study provided a novel insight into how strain gradients and electric polarization might interact to trigger osteocyte activity and accelerate bone healing. Although the study did not directly assess the biological effects of electric polarization, it estimated these effects indirectly through computational and radiographic analysis of strain changes that may trigger the bone healing process. The study highlighted a specific mechanical loading frequency capable of inducing strain within the optimal osteogenic window, addressing a gap in previous research that lacked clear rehabilitation protocols to trigger bone healing effectively without risking mechanical overload. This approach offered a potential non-invasive, cost-effective strategy with future applications in managing complex fractures and enhancing healing outcomes. The findings are expected to promote the early recovery of pediatric patients, enabling a faster return to normal activities, thus minimizing complications, and reducing the financial burden associated with extended care.

## 2 Methods

A computational observational study was conducted using finite element analysis to evaluate the biomechanical and electrophysiological effects on fracture healing. This was followed by an experimental cross-sectional validation study involving two children with greenstick fractures fracture angulation ≤20°, and the absence of osteoporosis or neurological conditions.^26^, ^27^ The two children were assigned based on nonprobability (convenience) sampling. one child (Child A, 9 years old) with right side greenstick fracture was treated conservatively using a splint, while the other (Child B, 10 years old) with right side greenstick fracture received electrical stimulation. Radiographic X-rays were collected for both children immediately after the trauma, one week after treatment initiation, and three weeks later to monitor fracture gap healing and assess gap strain. MATLAB was then utilized to process and analyze the X-ray images to calculate gap strain.^28^ The study was approved by the Ethical Committee of Cairo University (Approval Number: P.T.REC/012/005143) and registered with ClinicalTrials.gov under ID NCT06510595.

### 2.1 Modelling and simulation the effect of isometric brachioradialis muscle force

**Fig. 1:**
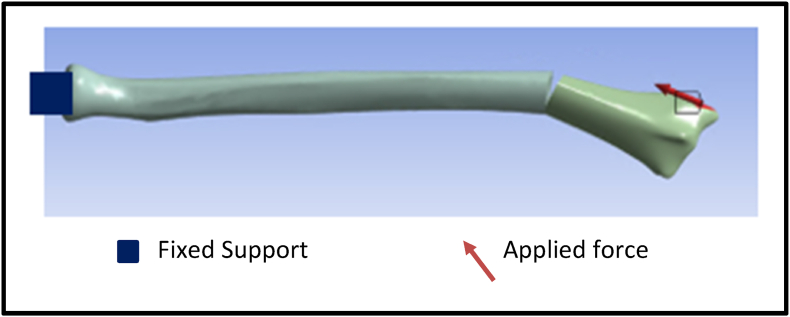
Simulation of isometric brachioradialis contraction during exercise, applying a horizontal force of 11 N, a vertical force of 2.5 N, and fixed support, generated using ANSYS software.

The finite element analysis (FEA) procedure involved creating a 3D radius bone model obtained from the GrabCAD online platform, with the fracture gap designed using SolidWorks 2023 (Dassault Systèmes). Static simulations were conducted in ANSYS (Workbench 2023) to evaluate the static mechanical effects of brachioradialis muscle force on fracture gap strain. Radius bone was fixed at the proximal end and muscle force applied at radial tuberosity (Fig. 1). The material property of bone was assigned using a modulus of elasticity of 7300 MPa, based on established data for pediatric bone.^29^, ^30^ The horizontal and vertical force components were set at 11 N and 2.5 N, respectively.^14^ The mean impulse per repetition was calculated by multiplying the applied force by the standardized repetition time of 1 s.^31^ The number of repetitions varied from 3 to 30, simulating different cumulative forces and their impact on strain and fracture healing. Consequently, the cumulative force exerted by the brachioradialis muscle ranged from 7.5 to 62.5 N vertically and 33–275 N horizontally, accurately reflecting muscle-induced loading during elbow flexion.

**Fig. 2:**
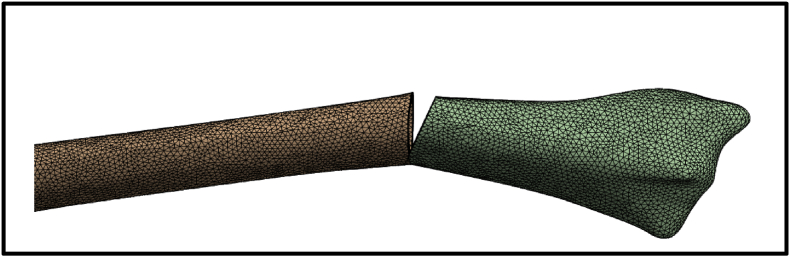
The mesh configuration with 295,601 nodes and 187,563 elements.

Then, the mesh was generated with a total of 295,601 nodes and 187,563 elements to balance computational efficiency and result accuracy, providing detailed insights into strain distribution across the fracture gap. Preliminary convergence tested by refining mesh size until fracture gap strain variation was <5 % between iterations (Fig. 2).

### 2.2 Simulation the effect of flexoelectricity on fracture displacement

Additionally, the flexoelectric effect was modeled using COMSOL Multiphysics 6.2 to study the flexoelectric effect on gap fracture healing^32^, ^33^, ^34^, ^35^ by incorporating the following parameters (Table 1).

**Table 1:** Mechanical and electrical properties of bone.^36^, ^37^, ^38^, ^39^

Parameter	Value
Modulus of elasticity (E)	114 × 10^9^ N/m^2^
Density (ρ)	0.366 kg/cm^3^
Characteristic length (L)	2.71 × 10^−9^ m
Electric susceptibility (χ)	15
Flexoelectric tensor (ε)	1.6 nC m^−1^

The applied electrical potential across the cortex was calculated based on the resistive properties of the hydrogel electrode, skin, muscle, and cortical layers. Resistance (R) for each layer was determined using formula R = ρ⋅d/A where ρ represents resistivity, d is thickness, and A is the cross-sectional area (Table 2). The total resistance was then used to calculate the electrical potential by applying Ohm's Law (V=IR) with an assumed current of 5 mA.^40^

**Table 2:** Material properties of hydrogel, skin, and cortical layers.^41^^,^^42^

Layer	Resistivity (Ω·m)	Area (m^2^)	Thickness
Electrode layer	200	0.0025	0.002
Skin	586.4	0.0025	0.002
Muscle	3	0.002	0.01
Cortex	50	40 × 10^−6^	0.001

**Fig. 3:**
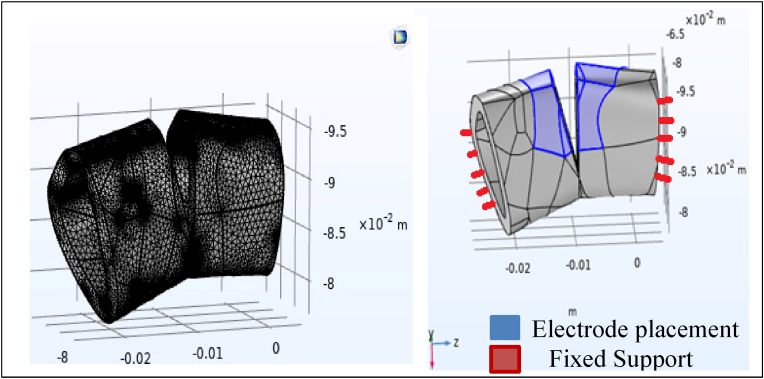
Finite element model illustrating the application of a 6.25 V electric potential across the fracture gap, including electrode placement, fixed support at the bone segment ends, and the meshing strategy with a maximum element size of 0.864 mm.

Subsequently, an electric potential of 6.25 V was applied across the fracture gap. The bone segment ends were fixed, and the finite element mesh was generated with a maximum element length of 0.864 mm (Fig. 3). Convergence testing confirmed mesh independence, with less than 5 % variation between iterations.

### 2.3 Experimental validation of fracture gap assessment using computational imaging

**Fig. 4:**
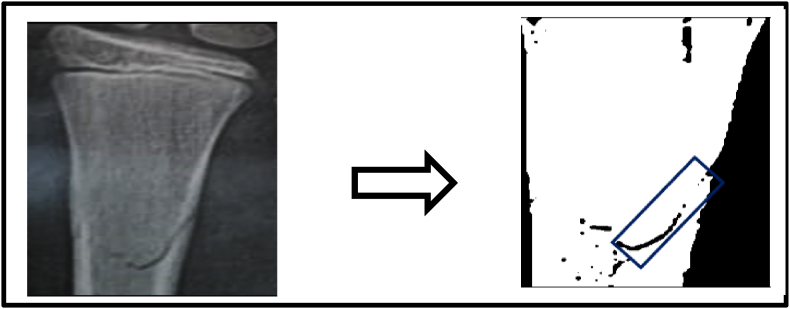
Grayscale and binarized X-ray image of the distal radius highlighting the fracture gap (black regions) for child A.

**Fig. 5:**
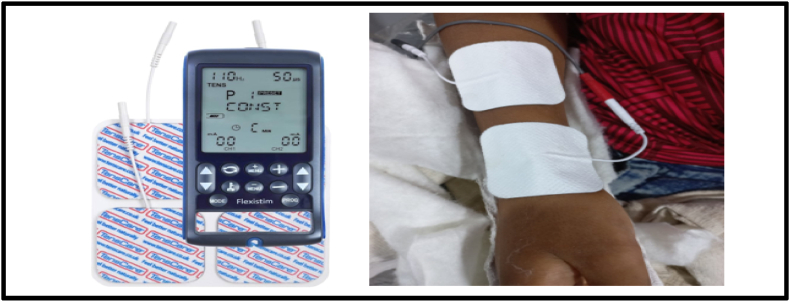
Electrode placement across the fracture gap in Child B for low frequency pulsed electrical stimulation. The setup delivered stimulation at 2 Hz with a pulse duration of 100 μs.

**Fig. 6:**
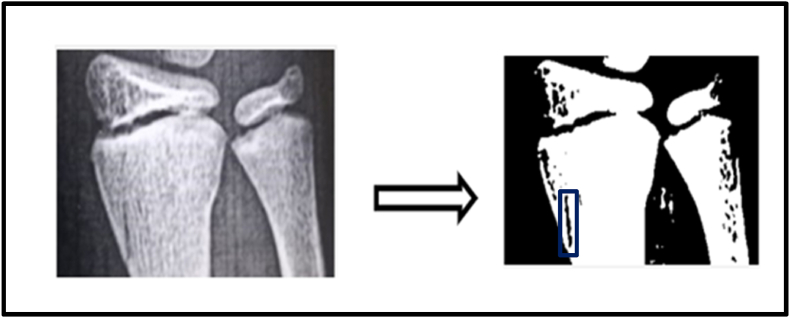
Grayscale and binarized X-ray image of the distal radius highlighting the fracture gap (black regions) for child B.

In order to validate the FEA model results, an experimental study was conducted on two children with greenstick fractures. Written informed consent was obtained from the parents of both children, and child assent was secured prior to participation. Child An underwent conservative treatment with a brace. Fracture gap strain was assessed after one week and three weeks using a computational approach. X-ray images of the distal radius were processed in MATLAB by converting them to grayscale and binarizing them to distinguish the fracture gap (black regions) from surrounding structures (Fig. 4). A polygonal region of interest (ROI) was manually selected to isolate the fracture site. The number of black pixels within the ROI, representing the fracture gap, was calculated, and the total gap area was measured in square centimeters. This method enabled quantitative evaluation of fracture gap healing. For Child B received electrotherapy using the Flexistim device (UK Rev 2.2 06/21) (Fig. 5), which delivered low-frequency pulsed stimulation at 2 Hz with a 100-μs pulse width during 30-min sessions. Self-adhesive electrodes were placed around the fracture site to ensure optimal signal transmission and coverage. The same MATLAB computational process was applied to evaluate the fracture gap area (Fig. 6), providing a comparative analysis of healing progress.^28^, ^29^

## 3 Results

### 3.1 Strain results from isometric muscle force

**Fig. 7:**
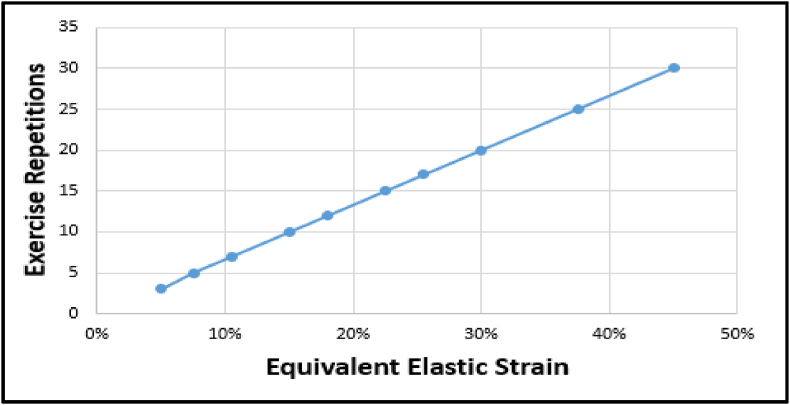
Linear increase in equivalent elastic strain (%) at the fracture gap with exercise repetitions. The strain was calculated to assess the mechanical response of the fracture site to cumulative isometric forces generated by the brachioradialis muscle, demonstrating a direct relationship between repetition number and strain magnitude.

The equivalent elastic strain at the fracture gap was analyzed to assess the mechanical response to isometric cumulative forces applied by the brachioradialis muscle. The results showed a linear relationship between the number of exercise repetitions and the strain at the fracture site (Fig. 7).

**Fig. 8:**
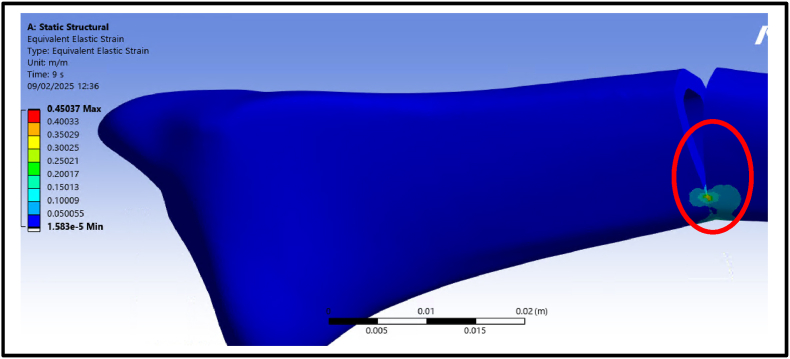
Strain distribution across the fracture gap simulated in ANSYS under static isometric loading. The figure illustrates how strain magnitude increases with exercise repetitions to a maximum of 45 % at thirty repetitions, highlighting the progressive mechanical deformation during brachioradialis muscle contraction.

The ANSYS simulations visually represented the strain distribution across the fracture gap (Fig. 8). At three repetitions, the strain was measured at 5 %, representing the lowest deformation. This value increased progressively with additional repetitions, reaching 8 % at five repetitions and 15 % at ten repetitions. The maximum strain of 45 % was observed at thirty repetitions. These results highlight how repetitive isometric loading increases strain at the fracture site, providing critical insights into the biomechanical response of the distal radius during muscle contraction.

### 3.2 Effects of electrical potential on the fracture gap

**Fig. 9:**
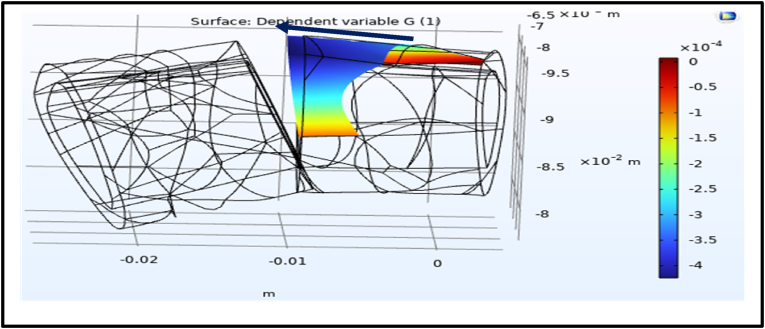
Shows the dependent variable strain (G) across the fracture gap. The blue color represents the strain magnitude about 4× 10^−4^ towards to the left side.

**Fig. 10:**
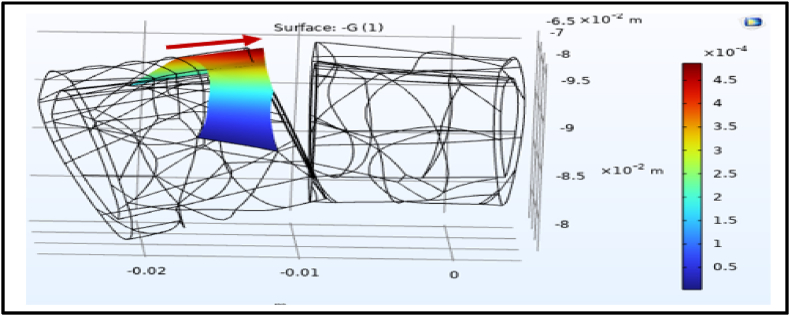
Shows the dependent variable strain (G) across the other fracture gap side. The red color represents the strain magnitude about 4× 10^−4^ towards to the right side.

The application of an electrical potential across the fracture gap was simulated using COMSOL Multiphysics to evaluate its impact on displacement and strain distribution. Under a voltage of 6.25 V, the simulations revealed a notable strain gradient induced by the electrical potential ranging between 4 × 10^−4^ and 4.5 × 10^−4^ (Fig. 9, Fig. 10).

### 3.3 Fracture gap area analysis before and after electrical stimulation

**Fig. 11:**
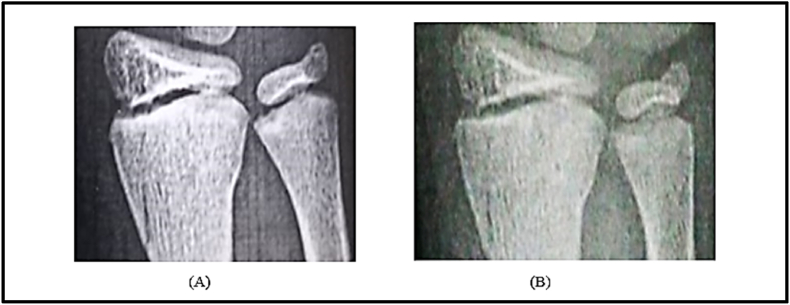
Comparison of fracture gap appearance: (A) after trauma and (B) one week post electrical stimulation application.

**Fig. 12:**
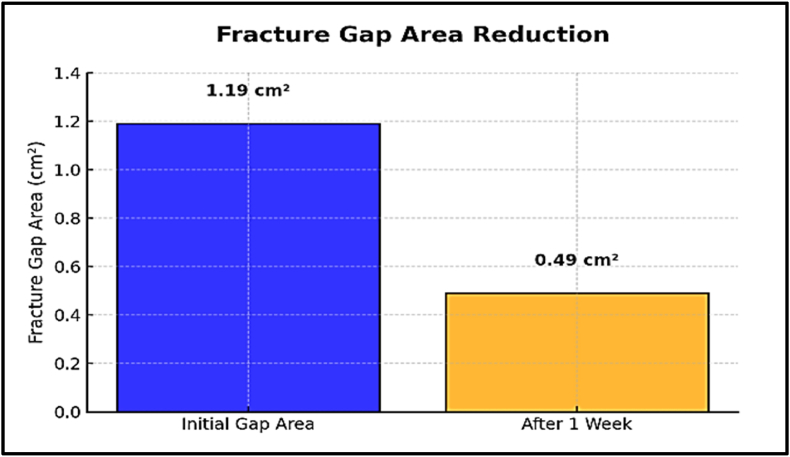
Quantitative comparison of fracture gap areas (cm^2^). (A) Initial fracture gap following trauma (1.19 cm^2^). (B) Reduced fracture gap area (0.49 cm^2^) after one week of electrical stimulation.

The fracture gap area was quantified before and after the application of electrical stimulation using MATLAB-based image analysis. Initially, the fracture gap area was calculated at 1.19 cm^2^. After one week of electrical stimulation application, the gap area reduced significantly to 0.49 cm^2^, representing a decrease of approximately 58.8 %. This reduction underscores the potential of electrical stimulation to promote fracture healing by accelerating the closure of the fracture gap (Fig. 11, Fig. 12).

### 3.4 Control case: conservative treatment

**Fig. 13:**
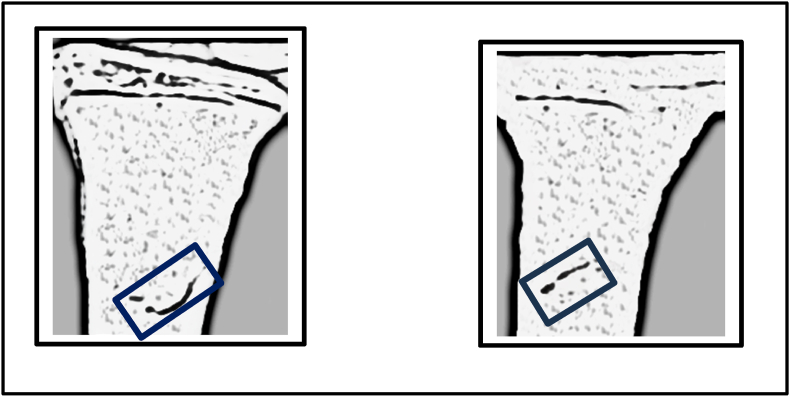
Fracture gap appearance before and after one week of conservative treatment with a brace in Child B.

**Fig. 14:**
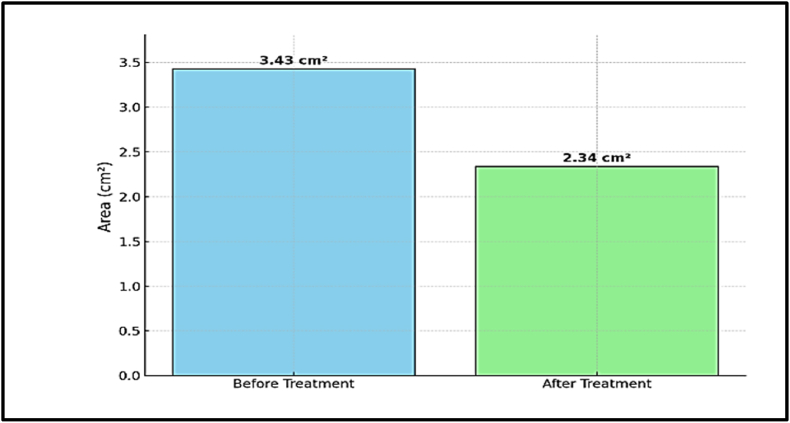
Quantitative comparison of fracture gap areas (cm^2^) before and after one week of conservative treatment. The initial area was 3.43 cm^2^, reducing to 2.34 cm^2^ after treatment.

The control case, Child B, underwent conservative treatment with a brace. The initial fracture gap area was calculated at 3.43 cm^2^. After one week, the fracture gap reduced to 2.34 cm^2^, showing a decrease of about 1.09 cm^2^ (31.8 %). This result demonstrated a slower rate compared to electrotherapy (Fig. 13, Fig. 14).

### 3.5 Comparative analysis of electrotherapy and conservative treatment after 3 weeks

**Fig. 15:**
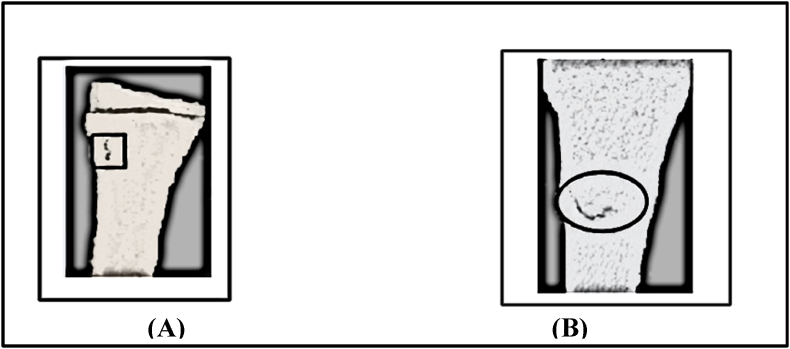
Comparison of X-ray images after three weeks of treatment. Child A (electrotherapy) shows significantly enhanced bone healing with advanced osseous consolidation and greater fracture gap reduction compared to Child B (conservative treatment), which demonstrates less pronounced healing.

A comparative analysis of fracture healing after three weeks revealed significant differences between the two treatment approaches. In the electrotherapy case (Child A), X-rays showed enhanced bone regeneration, evidenced by advanced osseous consolidation and a greater reduction in the fracture gap. Conversely, the conservative treatment case (Child B) displayed less pronounced healing relative to the other case (Fig. 15).

## 4 Discussion

Greenstick fractures, particularly in children, are a common forearm injury, with the distal third of the radius being the most frequently affected site. Conventional treatment often involves immobilization via either cast or an elasticated bandage for 4–6 weeks to allow adequate healing.^6^ However, this study explored innovative approaches to accelerate the healing process by evaluating the biomechanical and electrophysiological effects of applied controlled brachioradialis muscle force and electrical stimulation on greenstick distal third of the radius fracture healing using finite element analysis and experimental validation. The findings provided significant insights into the biomechanical and physiological processes influencing greenstick fracture healing.

The greenstick fracture population was selected for this study due to the ease of implementing therapeutic interventions during the healing phase. The removable nature of the detachable brace or Plaster Backslab allows for efficient application of physiotherapy modalities, including targeted exercises and electrotherapy, without disrupting immobilization or alignment. This flexibility ensures that the proposed interventions can be practically applied in clinical settings, making the findings of this study both relevant and applicable.

### 4.1 Mechanical forces and strain dynamics

The finite element analysis (FEA) revealed the critical role of the brachioradialis muscle in affecting fracture gap strain during isometric contractions. Under static loading conditions, repetitive muscle contractions resulted in progressive strain within the fracture gap. At three repetitions, the strain was measured at 5 %, while at 7 repetitions, it increased to 11 %. These findings align with Perren's strain theory, which emphasizes the importance of maintaining strain levels within a 2–10 % range to support callus formation and healing.^12^

Furthermore, the results suggested that controlled isometric exercises with moderate repetitions (3–5) can promote callus formation and enhance healing without exceeding the critical strain threshold. However, higher repetitions may introduce excessive mechanical stress, risking delayed healing or nonunion.^12^ This underscores the importance of optimizing rehabilitation protocols to balance mechanical stimulation with the biological demands of bone repair, particularly the fractures (greenstick) managed with splints, controlled interfragmentary motion is essential to facilitate secondary healing, as splints provide relative stability conducive to progressive mineralization. This result is supported by the piezoelectric and flexoelectric properties of cortex, which generate electrical charges when deformed, especially at crack tips, enhancing osteocyte activity and promoting bone remodeling, indicating that these electrical properties help in optimizing the bone repair process, encouraging cell activity and improving the healing response.^18^, ^19^

### 4.2 Electrical stimulation and bone healing

Electrical stimulation has been explored for its potential to enhance fracture healing through converse flexoelectric findings, which demonstrate that strain gradients in bone can generate electric potentials influencing osteocyte activity, angiogenesis, and bone mineralization, leading to significantly faster healing times in distal radius and bone formation. Thus, converse flexoelectricity may represent a natural and complementary mechanism underlying the beneficial effects observed with external electrical stimulation therapies, such as pulsed electromagnetic fields in fracture healing.^43^^,^^44^ Furthermore, the converse flexoelectric effect was investigated using COMSOL Multiphysics to evaluate the influence of electrical potential on fracture healing. Applying a 6.25 V electrical potential across the distal radius fracture gap resulted in a strain gradient ranging from 400 to 450 με which is below the 2 % threshold considered critical for optimal bone healing. On the other hand, the experimental validation using Electrical Stimulation demonstrated significant progress in healing, with a 58.8 % reduction in fracture gap area after one week compared to the control one. This highlighted the potential of electrical stimulation as a non-invasive intervention for enhancing bone repair. The experimental results are further supported by the concept of converse flexoelectricity, where the mechanical strains produced by this phenomenon activate osteocytes, highlighting its potential as a therapeutic tool to promote bone repair.^20^ Additionally, electrical stimulation enhances bone healing both chemically and physically. Chemically, at the cathode (negative electrode), it produces hydroxyl ions that increase pH and reduce oxygen levels, which enhances osteoblast activity (bone formation) and suppresses osteoclast activity (bone resorption). The production of hydrogen peroxide further stimulates osteoclast differentiation and increases VEGF secretion, promoting angiogenesis. Moreover, it elevates levels of bone morphogenetic proteins (BMPs 2, 6, and 7) essential for bone formation.^45^ Physically, osteocytes, embedded within the mineral matrix, are highly sensitive to local electric and strain fields, which regulate their signaling for bone formation or resorption.^46^ The minor computational strain results can be attributed to the oscillating nature of electrical stimulation, which may not provide the stable electrochemical environment needed for optimal bone remodeling. In contrast, previous studies elaborated that Direct Current Electrical Stimulation (DCES) generates a consistent electric field that has been shown to enhance osteoblast differentiation, angiogenesis, and calcium signaling, all of which are critical for promoting bone repair. These characteristics of DCES suggest it may be more effective in supporting fracture healing. Future studies should investigate the potential advantages of DCES over electrical stimulation in optimizing fracture healing outcomes.^47^, ^48^

### 4.3 Limitations

While the findings provided valuable insights, several limitations should be acknowledged. The computational mechanical simulations relied on theoretical models and lacked an experimental validation for the mechanical strain results. Furthermore, it is recommended for future randomized controlled trials to compare different exercise protocols to confirm these findings in clinical settings. The experimental application of electrical stimulation demonstrated limited mechanical effects at the tested settings, suggesting that the parameters used may not have been optimal for inducing significant strain. Future research should investigate higher voltages or alternative electrical stimulation methods, such as DCES, to evaluate their impact on strain distribution and healing outcomes. Additionally, the FEA and COMSOL simulation results were not validated against experimental strain gauge measurements or cadaveric mechanical testing. Future studies should include experimental mechanical validation to strengthen model accuracy and clinical applicability. Besides that, image analysis for fracture gap area faced challenges due to the presence of the splint, which blurred the X-ray images, reducing the accuracy of binary conversion. Improved imaging techniques or advanced computational tools are recommended to enhance precision in future studies. Additionally, the small sample size in this study limits the generalizability of the findings. A larger cohort is essential with more controlling the confounding variables, including the fracture displacement differences to minimize the individual healing variability and strengthen the statistical validity and broader applicability of the results.

## 5 Conclusion

This study demonstrates that applying controlled isometric loading and electrical stimulation may help reduce the fracture gap in greenstick distal radius fractures, potentially accelerating healing. However, these findings are preliminary and exploratory aiming to model potential electric field effects rather than replicate exact in vivo biological responses. Further randomized controlled trials with larger sample sizes are required to validate these results and establish clinical efficacy.

## CRediT authorship contribution statement

**Mohamed Hassan:** Conceptualization of mechanical fracture modelling and simulation; research design; methodology; data analysis; manuscript preparation. **Enas Fawzi Youssef:** Refinement of the research approach, critical feedback, revision of the manuscript. **Ahmed Rizk Mohamed:** Clinical monitoring of participants, provision of follow-up data post-trauma. **A.R. El-Dhaba:** Conceptualization, of the flexoelectric effect; mathematical simulation, Methodology. **Mohamed I. Zineldin:** Conceptualization, implementation of computational MATLAB. **Dina S. Abd Allah:** Guidance on research design, Methodology, data interpretation, critical revision to enhance manuscript quality.

## Data availability statement

All data generated or analyzed during this study are available from the corresponding author upon reasonable request.

## Guardian/patient consent statement

Written informed consent was obtained from the parents or legal guardians of all child participants included in this study, in accordance with ethical guidelines and institutional requirements.

## Ethical statement

This study involved children participants and was conducted in accordance with the ethical standards of the institutional and national research committees. Ethical approval was obtained from the appropriate ethics committee, and written informed consent was secured from the parents of all participating children.

## Funding statement

This research did not receive any specific grant from funding agencies in the public, commercial, or not-for-profit sectors.

## Declaration of competing interest

The author declares that there is no conflict of interest regarding the publication of this paper.
